# Venous thromboembolism and mortality in breast cancer: cohort study with systematic review and meta-analysis

**DOI:** 10.1186/s12885-017-3719-1

**Published:** 2017-11-10

**Authors:** Umair T. Khan, Alex J. Walker, Sadaf Baig, Tim R. Card, Cliona C. Kirwan, Matthew J. Grainge

**Affiliations:** 1 Division of Epidemiology and Public Health, School of Medicine, University of Nottingham, Medical School, Nottingham, NG7 2UH UK; 2 Institute of Translational Medicine, Molecular and Clinical Cancer Medicine, University of Liverpool, Crown Street, Liverpool, L69 3BX UK; 3 School of Life Sciences, University of Nottingham, Medical School, Queen’s Medical Centre, Nottingham, NG7 2UH UK; 4 Institute of Cancer, University of Manchester, South Manchester University Hospitals NHS Trust, Wythenshawe Hospital, Southmoor Road, Manchester, M23 9PL UK

**Keywords:** Breast cancer, Venous thromboembolism, Pulmonary embolism, Deep vein thrombosis, Mortality, Prognosis, Cohort study, Systematic review, Meta-analysis

## Abstract

**Background:**

Breast cancer patients are at an increased risk of venous thromboembolism (VTE). However, current evidence as to whether VTE increases the risk of mortality in breast cancer patients is conflicting. We present data from a large cohort of patients from the UK and pool these with previous data from a systematic review.

**Methods:**

Using the Clinical Practice Research Datalink (CPRD) dataset, we identified a cohort of 13,202 breast cancer patients, of whom 611 were diagnosed with VTE between 1997 and 2006 and 12,591 did not develop VTE. Hazard ratios (HR) were used to compare mortality between the two groups. These were then pooled with existing data on this topic identified via a search of the MEDLINE and EMBASE databases (until January 2015) using a random-effects meta-analysis.

**Results:**

Within the CPRD, VTE was associated with increased mortality when treated as a time-varying covariate (HR = 2.42; 95% CI, 2.13–2.75), however, when patients were permanently classed as having VTE based on presence of a VTE event within 6 months of cancer diagnosis, no increased risk was observed (HR = 1.22; 0.93–1.60). The pooled HR from seven studies using the second approach was 1.69 (1.12–2.55), with no effect seen when restricted to studies which adjusted for key covariates.

**Conclusion:**

A large HR for VTE in the time-varying covariate analysis reflects the known short-term mortality following a VTE. When breast cancer patients are fortunate to survive the initial VTE, the influence on longer-term mortality is less certain.

**Electronic supplementary material:**

The online version of this article (10.1186/s12885-017-3719-1) contains supplementary material, which is available to authorized users.

## Background

Breast cancer is the most common type of cancer amongst women worldwide accounting for approximately 1.67 million new cases and 522,000 deaths in 2012 [1], and therefore imposes a considerable disease burden on healthcare resources across the globe. The association between cancer and venous thromboembolism (VTE) which includes deep vein thrombosis (DVT) and pulmonary embolism (PE) was first established more than 10 decades ago by Trousseau [2]. A developing body of evidence indicates changes in the hemostatic system even when VTE is absent in cancer patients, with a symbiotic relationship between the hemostatic system and tumour cells [3].

It is reported that breast cancer patients are 3–4 fold more likely to develop VTE compared with patients of equivalent age without cancer [4, 5]. Our recent work [6] and other studies [7–9] have shown that this risk is accentuated further in breast cancer patients receiving tamoxifen and chemotherapy up to 5-fold and 10-fold, respectively. The association between the development of VTE in patients with cancer and reduced overall survival was first evidenced in a seminal paper published in 2000 by Sorensen and colleagues which found that the 12-month survival rate was 3-times higher in cancer patients without a VTE [10]. Subsequent research has reported similar findings for a variety of specific cancer types suggesting that VTE could potentially be used a marker for severe and more aggressive forms of cancers [11–14]. Relevant data specific to women with breast cancer, however, are still lacking.

VTE associated with breast cancer is a devastating complication, which occurs among women with an otherwise good health prognosis. By establishing the extent to which a VTE influences prognosis, especially longer-term, the implications of both prophylactic and therapeutic anticoagulation on preventing mortality can be more fully understood. We therefore present new data from a UK based cohort study and pool this with existing published and unpublished data in a systematic review and meta-analysis to assess the risk of mortality in breast cancer patients with VTE compared to those without VTE.

## Methods

A summary of this was work previously published as a poster at the National Cancer Research Institute conference in 2015 [15].

### Cohort study (clinical practice research Datalink, CPRD)

#### Study population

The study includes data from the CPRD, previously known as the General Practice Research Database, until April 2013. It contains population-based electronic health data on about 8% of the UK population [16] which has been prospectively collated from over 600 GP practices in the UK from 1987 onwards. It is an anonymous database, which collects information on patient demographics, clinical diagnoses, treatments and outcomes amongst other variables. Its population is considered to be broadly representative of UK population in terms of age and sex structure [17] and its quality and completeness has been validated in various studies [18, 19]. Use of these data was approved by the CPRD Independent Scientific Advisory Committee (ISAC, protocol number- 10_091). ISAC is a non-statutory expert advisory body which provides a formal review for requests to access data from the CPRD.

The data used in this paper are based on about 50% of CPRD practices in England for which the data is linked to the following: Hospital Episodes Statistics (HES), providing information on primary and secondary diagnoses and inpatient procedures; National Cancer Intelligence Network (NCIN), providing information on cancer diagnoses; and Office of National Statistics (ONS), providing information on dates and underlying causes of death. We selected all women with a first breast cancer diagnosis (ICD-10 code C50) using just the NCIN (cancer registry) source from 1st April 1997 (the date from which linked data were first available) until 31st December 2006. These patients were followed up until they died, left a participating CPRD practice or 31st December 2010, whichever came first. We excluded women who were i) under 18 years old at the time of diagnosis, ii) diagnosed in the 1st year of registration at a participating CPRD practice; iii) diagnosed with breast cancer outside the CPRD and HES registration periods; iv) developed VTE prior to first cancer diagnosis.

#### Exposure, outcome and covariates

VTE was established when a medical code for venous thromboembolism (ICD 10; I26, I80-I82) in either or both the CPRD and HES was supported by evidence of an anticoagulant prescription or medical code providing evidence of anticoagulation being recorded between 15 days before and 90 days after the VTE event date. Only the first VTE event following the cancer diagnosis was considered in this study. This algorithm for defining VTE has been previously validated using primary care data alone [20]. Information on all deaths, including dates of death, were established from the linked ONS mortality data which were available for all women in the study cohort. Covariates included cancer stage which was classified as either “local disease” (confined to the breast), “regional disease” (axillary lymph node involvement), “distant metastases” (any evidence of distant metastases) or “unknown stage”. An individual comorbidity score excluding breast cancer (Charlson score) was calculated from GP records and coded into three levels (0,1–3,≥3). Other covariates (age, smoking status, BMI, surgery, chemotherapy and endocrine therapy) are defined in exactly the same way as in our previous paper from this cohort [6].

#### Statistical analysis

Multivariate cox adjusted proportional hazard ratios were calculated for the VTE group compared to control group using ‘STATA 13’. The survival analysis was conducted using time-varying covariate (TVC) analysis where VTE status changed from “unexposed” to “exposed” at the time a VTE was diagnosed to ensure hazard ratios gave an accurate representation of the risk of mortality as the patients’ VTE status changed. Survival analysis started at the time of breast cancer diagnosis for all women. A non-time-varying covariate analysis (nTVA) was also conducted where women assumed the same “exposure level” throughout the entire follow-up period. Patients who developed VTE in the first 6 months after diagnosis of breast cancer were defined as the VTE group and these were compared with women who did not develop VTE. Any woman who died in this 6 month exposure period was excluded from the nTVA analysis. This approach referred to as the “Landmark” approach [21] has the advantage of excluding the potential for immortal time bias [22]. Follow-up commenced at the end of the 6 months exposure window, and subsequent mortality in the VTE and non-VTE groups was compared using a cox proportional hazards model. Both types of analysis (TVC and nTVA) were adjusted for age, stage, grade, comorbidity, tamoxifen treatment, smoking, body mass index, surgery and chemotherapy.

### Systematic review and meta-analysis

#### Data sources and searches

This review was carried out and reported in line with the Preferred Reporting Items for Systematic Reviews and Meta-Analysis (PRISMA) guidelines for the reporting of clinical trials and observational studies [23]. A comprehensive search of OVID MEDLINE from 1946 to January week 1, 2015 and EMBASE from 1974 to January week 2, 2015 was carried out to identify published cohort studies and conference abstracts (EMBASE only) which provided survival data on breast cancer patients with VTE (Additional file 1: Appendix 1). Search terms relating to breast cancer and venous thromboembolism were adapted from previous Cochrane Collaboration reviews [24–26] and our earlier systematic review on cancer and thrombosis [27] whilst Scottish Intercollegiate Guidelines Network (SIGN) validated terms were used as a filter for observational studies in MEDLINE [28].

#### Study Selection.

Titles, abstracts and full texts were independently reviewed by two authors; AJW, SB for MEDLINE studies identified up until October 2012 and UTK, MJG for studies identified via EMBASE and in an updated MEDLINE search carried out in January 2015. Any discrepancies in decision for inclusion or exclusion of a particular paper were resolved by mutual discussion amongst the authors. The following criteria were used in the inclusion and exclusion of papers:

Study Design: All cohort studies (retrospective and prospective) published as either full text articles or published conference proceedings in the English language were considered for inclusion. Where data appeared in the form of a published abstract from a conference (within EMBASE), they were assessed for inclusion in the same way as published journal articles. Authors of conference abstracts judged as being of relevance were contacted in an attempt to obtain additional information both to determine potential inclusion of the study and obtain unpublished data if it transpired the study met our inclusion criteria. Data from randomised-controlled trials (RCTs) were excluded from selection as it is not recommended practice to combine data from observational studies and RCTs [29] and since RCTs may not be representative of all cancer patients with or without VTE as they usually contain a select group of patients [30].

Participants: Studies containing women (18 years old and above) with breast cancer were considered. Studies containing patients with a mixture of cancer types were excluded unless data were presented separately for women with breast cancer. There were no restrictions made on the basis of nationality or stage of disease.

Exposure: Studies with breast cancer patients who had defined VTE as an exposure group were considered. Studies where all patients had or developed VTE were excluded as it would not be possible to explore the impact of a VTE on mortality in this instance. VTE was defined as patients with deep vein thrombosis (DVT), pulmonary embolism (PE). Other types of VTE, such as portal vein thrombosis and vena cava thrombosis were included if data were combined with DVT and PE. We did not include VTE events associated with venous-catheter use so as not to introduce further heterogeneity (as prognosis following these is likely to be different).

Outcome: The outcome was all cause mortality. Survival data were only considered if papers presented hazard ratios or Kaplan-Meier graphs comparing survival data between breast cancer patients with VTE (cases) and breast cancer patients without VTE (controls).

#### Data extraction

Data extraction was performed independently by two reviewers (either SB, MJG or UTK, MJG). For the instance where hazard ratios were estimated from a Kaplan-Meier plot, this was done independently using the formula developed by Parmar et al. [31]. The average readings of the two survival probabilities for the two reviewers at each time point was taken when discrepancies occurred. Where data were presented in the form of hazard ratios, the standard error was calculated for hazard ratios from each paper using upper and lower confidence intervals.

#### Statistical analysis

Hazard ratios were pooled under the assumption of random effects [32] using ‘STATA 13’. Separate pooling of results was carried out for studies conducting TVC analysis, where women changed from non-exposed to exposed at the time they develop VTE during survival follow-up and nTVA, where exposure groups were defined in the beginning of the study and women remained in the same group throughout follow-up. Sub-group analyses were performed on studies, which conducted nTVA to address heterogeneity: (1) Whether studies were adequately adjusted for key confounders; (2) Whether VTE occurred before or after cancer diagnosis. With regards to (1), a study was judged to be adequately adjusted if it adjusted for at least two of the three covariates: (i) age, (ii) co-morbidity and/or performance status, (iii) stage of breast cancer. Studies that did not meet the criteria were classed as ‘non-adjusted’. With regards to (2), where the VTE event occurred before cancer diagnosis for the majority of patients in the study; these studies were grouped together and compared with studies where patients developed VTE after cancer diagnosis to enable us to explore whether the time when the patients develop VTE influences mortality. Equivalent sub-group analyses were not presented for studies conducting a TVC analysis due to the small number of studies (*n* = 2) and homogeneity of results between these. Heterogeneity was assessed using the I-square statistic in all instances.

## Results

### Cohort study (CPRD)

#### Study population

From the CPRD database, a total of 13,202 patients with a new diagnosis of breast cancer were identified. In total, 611 women developed VTE during the study period (cases) and these were compared with 12,591 women who remained free from VTE (controls). The median age was 62 years (IQR 52–74) and 3.6% of women with VTE had distant metastases compared with 3.4% of those without VTE (the corresponding figures with disease localized to the breast with no nodal involvement were 38.3% and 35.2%; respectively). In total, 3504 (27.8%) women in the control group died during the study period compared with 298 (48.8%) in the VTE group. A comparison of the groups is summarized in Table 1.

**Table 1:** Summary of patient characteristics from the CPRD

Total		No VTE	%	VTE	%
		12,591		611	
Cancer stage	Local disease	4823	38.3	214	35
	Regional disease	2800	22.2	161	26.4
	Distant metastases	449	3.6	21	3.4
	Unknown	4519	35.9	215	35.2
Age (years)	<40	694	5.5	21	3.4
	40–49	1967	15.6	63	10.3
	50–59	3154	25	108	17.7
	60–69	2794	22.2	179	29.3
	70–79	2249	17.9	158	25.9
	80–89	1733	13.8	82	13.4
Charlson score	0	6692	53.1	295	48.3
	1 to 3	5567	44.2	293	48
	≥4	332	2.6	23	3.8
Current Smoking	No	11,602	92.1	572	93.6
	Yes	989	7.9	39	6.4
Body mass Index (kg/m^2^)	Underweight (<19)	188	1.5	4	0.7
	Ideal (19.0–24.9)	3006	23.9	93	15.2
	Overweight (25.0–29.9)	2372	18.8	148	24.2
	Obese (30.0–34.9)	1046	8.3	73	11.9
	Morbidly obese (≥35.0)	402	3.2	38	6.2
	Missing	5577	44.3	255	41.7
Surgery	No	2989	23.7	104	17
	Yes	9602	76.3	507	83
Chemotherapy	No	10,007	79.5	422	69.1
	Yes	2584	20.5	189	30.9
Endocrine therapy	No	2232	17.8	87	14.2
	Yes	10,316	82.2	524	85.8
Died	No	9087	72.2	313	51.2
	Yes	3504	27.8	298	48.8

#### Survival analysis

Overall, the crude hazard ratio (HR) was 2.97 (95% CI 2.62–3.36) in the analysis where VTE was treated as a time-varying covariate. The HR was 2.42 (95% CI 2.13–2.75) after adjustment for covariates (Table 2). For patients with earlier stage of disease, the relative influence of VTE on mortality was greater compared with those for whom the disease had spread (adjusted HR 2.94 (95% CI 2.29–3.77 for local disease, 2.53 (95% CI 2.01–3.19) for regional disease (axillary node involvement) and 1.47 (95% CI 0.82–2.63) for distant metastases. When results were stratified by comorbidity score into three levels (Charlson score 0, 1–3, ≥4) there was no notable difference in the magnitude of the HRs between the three subgroups (Additional file 2: Table S1).

**Table 2:** Results from CPRD (time-varying and non-time-varying covariate analysis by adjustment)

Time-Varying (follow-up from cancer diagnosis)												
				Unadjusted			Adjusted for age			Multivariate Model^a^		
		No. of patients	No. Died	HR	95% CI		HR	95% CI		HR	95% CI	
All patients	No VTE	12,591	3504	1			1			1		
	VTE	611	298	2.97	2.62	3.36	2.58	2.27	2.92	2.42	2.13	2.75
Local disease	No VTE	4823	726	1			1			1		
	VTE	214	81	4.05	3.17	5.16	3.21	2.51	4.1	2.94	2.29	3.77
Regional disease	No VTE	2800	798	1			1			1		
	VTE	161	89	3.23	2.57	4.05	2.9	2.31	3.64	2.53	2.01	3.19
Distant metastases	No VTE	449	300	1			1			1		
	VTE	21	14	1.4	0.8	2.47	1.27	0.72	2.24	1.47	0.82	2.63
Unknown stage	No VTE	4519	1680	1			1			1		
	VTE	215	114	2.45	2	3.01	2.27	1.85	2.78	2.33	1.89	2.87
Non-time-varying (follow-up commencing 6 months after cancer diagnosis)												
All patients	No VTE	12,148	3171	1			1			1		
	VTE	138	54	1.63	1.24	2.14	1.50	1.15	1.96	1.22	0.93	1.60
Local disease	No VTE	4854	743	1			1			1		
	VTE	45	11	1.72	0.95	3.12	1.51	0.83	2.74	1.25	0.68	2.27
Regional disease	No VTE	2834	834	1			1			1		
	VTE	46	22	1.73	1.13	2.64	1.53	1	2.34	1.17	0.76	1.79
Distant metastases	No VTE	331	212	1			1			1		
	VTE	5	3	1.34	0.43	4.18	1.37	0.44	4.3	1.32	0.40	4.35
Unknown stage	No VTE	4129	1382	1			1			1		
	VTE	42	18	1.37	0.86	2.18	1.34	0.84	2.14	1.23	0.77	1.96

^a^age plus: stage (where not stratified), grade, comorbidity, tamoxifen therapy, smoking, body mass index, surgery and chemotherapy. In the time-varying analysis, no. died represents the number of deaths in women who never developed VTE

For the non-time varying covariate analysis (Table 2) the unadjusted HR was significant, 1.63 (95% CI 1.24–2.14), however after adjustment for the same covariates listed above, this became non-significant, 1.22 (95% CI 0.93–1.60). Subsequent subgroup analysis for the various stages of breast cancer reported no significant difference in mortality between women with and without VTE in any of the four subgroups (Table 2). The relationship with mortality to the other covariates in these data is summarised in Additional file 3: Table S2.

### Systematic review and meta-analysis

#### Selection of studies

**Fig. 1:**
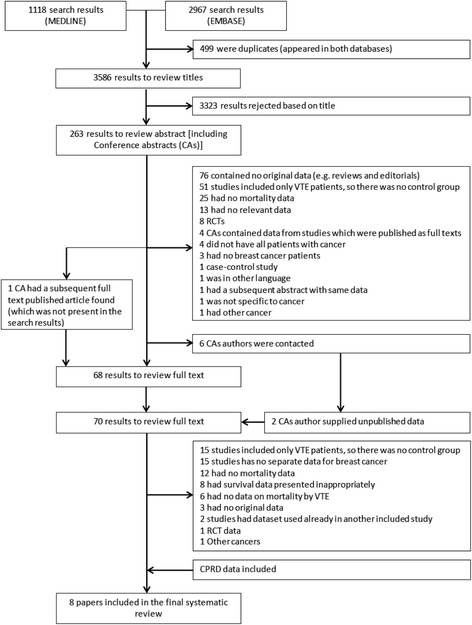
Summary of search results and breakdown at each stage. CA conference abstracts

A total of 4085 search results were generated from our search strategy and subsequently full text was obtained for 70 articles. Out of a total of 70 full text articles, 8 were selected for the final review with the addition of the CPRD data described above (Fig. 1). At the full text stage, there were 15 studies which would have met the inclusion criteria, except that they did not provide separate data on breast cancer patients. There were an additional 8 studies which met the inclusion criteria except that the survival data were presented in such a way that hazard ratios could not be estimated. Two studies published in the form of conference abstracts met all criteria for inclusion (from a total of 6 authors contacted), from which the authors supplied unpublished data, and provided consent for their data to be included in the study [33, 34].

#### Overview of included studies

Characteristics of individual included studies are in Additional file 4: Table S3. Overall, from the 8 included studies, 4 were from UK, 2 from USA, and 1 from Mexico and 1 from Brazil. Average age (median or mean) from the included studies ranged from 51 to 75 years. The median follow-up of studies (where available) ranged from 15.4 to 26.2 months. Two studies ([35]; CPRD) used a TVC analysis whereas the rest used nTVA. Out of the studies using nTVA, 3 studies ([9, 36]; CPRD) were adequately adjusted whereas 4 studies [33, 34, 37, 38] were classified as unadjusted as they did not meet our criterion for adjustment even though some studies had adjusted for other covariates [33, 37]. Furthermore, from the nTVA group, 5 studies defined VTE as occurring after cancer diagnosis ([9, 33, 34, 38]; CPRD) and 2 studies [36, 37] defined VTE occurring prior to diagnosis.

#### Random-effects meta-analysis

**Fig. 2:**
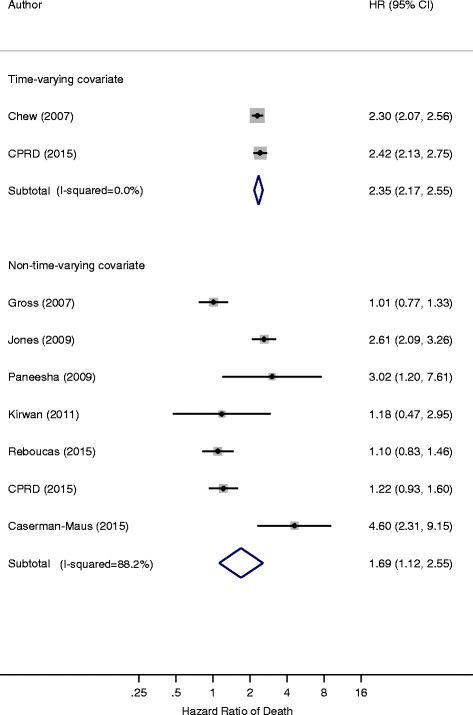
Forest plot of the hazard ratios by type of analysis, time-varying covariate compared to non-time-varying

When results from our cohort (CPRD) were pooled with one other study [35] which treated VTE as a TVC, the pooled HR for risk of mortality in breast cancer patients with VTE was 2.35 (95% CI 2.17–2.55) and heterogeneity was minimal. In a pooled analysis of results from seven studies (including the CPRD), which utilized nTVA, the overall hazard ratio was 1.69 (95% CI 1.12–2.55), however, heterogeneity was substantial (I-square = 89%, Fig. 2).

**Fig. 3:**
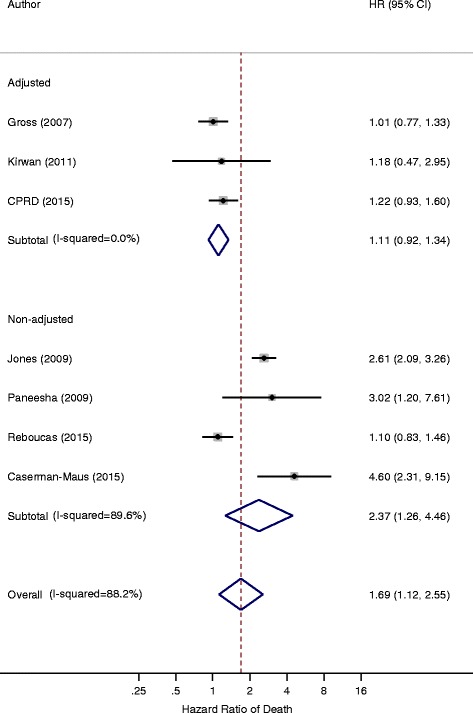
Forest plot of the hazard ratios of nTVA studies comparing adjusted to non-adjusted studies

The pooled HR from 4 studies which were unadjusted (or inadequately adjusted) was 2.37 (95% CI 1.26–4.46), in contrast to the 3 studies which had adequately adjusted for covariates, no increase in mortality was observed among patients with VTE [HR 1.11 (95% CI 0.92–1.34)], highlighting that the risk of mortality in breast cancers due to VTE was non-significant when adjusted for important covariates including age, stage and comorbidity (or performance status) (Fig. 3).

**Fig. 4:**
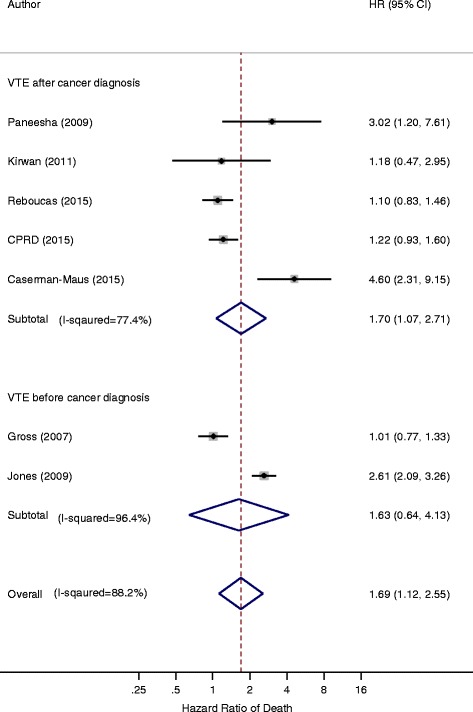
Forest plot of the hazard ratios of nTVA studies comparing ‘VTE before cancer diagnosis’ with ‘VTE after cancer diagnosis’

A second sub-group analysis was carried out on studies using nTVA by whether VTE occurred prior to cancer diagnosis or after it. The pooled HR for the 5 studies defining VTE after cancer diagnosis was 1.70 (95% CI 1.07–2.71) compared to the 2 studies which defined VTE before cancer diagnosis [HR 1.63 (95% CI 0.64–4.13)] (Fig. 4).

## Discussion

### Summary of findings

Based on data from a large cohort of women with breast cancer representative of the United Kingdom, the risk of mortality was more than doubled in the time following a VTE event, reflecting the high short-term mortality following a thromboembolic event. In contrast, using the landmark approach which assigned women as being a VTE or non-VTE case for the entire follow-up period, VTE exerted no increased risk of mortality once important covariates such as stage of disease and a measure of overall health status was taken into account. When our data were pooled with those from seven additional studies (including two which are currently unpublished), the pooled hazard ratio was 2.35 (2.17–2.55) for studies using a TVC analysis and 1.69 (1.12–2.55) for those using an nTVA, the latter of which contained substantial heterogeneity. The hazard ratio we report for TVC analysis is comparable to that reported by Posch et al. more recently of 2.98 (2.36–3.77) using a multi-state model applied to data from the Vienna Cancer and Thrombosis Study which considered all cancer types rather than breast cancer specifically [39]. Sub-group analyses reported higher HRs in studies which did not adjust for key covariates, whereas the timing of VTE diagnosis in relation to the cancer diagnosis did not have an appreciable impact on the magnitude of the hazard ratios observed.

### Strengths and limitations of the research

To our knowledge, this is the first attempt to systematically evaluate all available data exploring whether or not among women with breast cancer, the risk of mortality is raised following development of a VTE. Our systematic review was strengthened by inclusion of two established databases (MEDLINE and EMBASE) with carefully selected search terms. Furthermore, through obtaining additional data for studies originally published in the form of conference abstracts, we were able to include data which is currently unpublished in our synthesis of the evidence. Thirdly, by inclusion of our data from the CPRD we were able to include data in the overall synthesis which has the strength of utilizing recently linked primary, secondary and cancer registration data from a large representative sample of women from the UK. Our two distinct approaches to analysis, enabled us to assess the effect of a VTE on short-term and long-term mortality separately.

Limitations of our work include the fact the methods of meta-analysis employed in our systematic review relied on survival data being presented both separately for breast cancer patients in studies where patients with a mixture of cancer types were reported, and also in an appropriate numerical form so that hazard ratios (and standard errors or confidence intervals) from these could be obtained. As such there were several potentially relevant studies which have been conducted but which we were unable to include. Our systematic review also contained a high degree of heterogeneity, meaning that it was not possible for us to determine the “true” degree which developing a VTE has on subsequent mortality. Instead effect sizes would be influenced by characteristics of the study population (age, tumour characteristics and treatment modalities), methods for establishing VTE (including whether methods such as a Doppler scan were used to confirm the diagnosis) and duration of follow-up. In part, we were successful in elucidating specific reasons for this heterogeneity, namely that our finding in the CPRD that effect sizes were attenuated considerably after adjustment for key covariates was also demonstrated within one of the papers included in our systematic review [36]. However, even in sub-group analyses whereby data were stratified by factors, which we anticipated, would account of heterogeneity of results between studies, considerable residual heterogeneity remained in many instances (as indicated by the I-square statistic). Finally, our findings could be influenced by the potential for publication bias as is inherent with any systematic review. However, in the present review no obvious differences were found in the magnitude of the effect size between the five studies currently published and three presently unpublished.

Differences in methodological quality of original studies represent another potential source of heterogeneity in reviews of observational studies as addressed by the sub-group analyses described above. Similarly, methodological deficiencies in some or all of the component studies could bias estimates of the pooled result. Many of the source studies relied on routinely collected administrative data for determining VTE status in study participants. Misclassification of VTE events could attenuate the magnitude of an association between VTE and survival. In the CPRD, our algorithm for defining VTE was previously shown to have positive predictive value of 84% when compared with more detailed investigations of patient records [20]. However, this algorithm has not been validated specifically in cancer patients and would not capture anticoagulant prescriptions emanating from secondary care. In studies which did not use a TVC approach, the complex nature of the chronology between diagnosis of VTE, diagnosis of cancer and subsequent outcome could influence findings. For example, it is common for studies to start follow-up at the time of cancer diagnosis. If VTE occurs after this date then there would be a period of guaranteed follow-up time between the cancer and VTE dates, which would create a favourable impression of survival in this “exposed” group and thus weaken any true association (immortal time bias). Whilst we attempted to stratify results by timing of VTE and cancer, this information could not always be adequately established from the original study reports.

The potential for immortal time bias was avoided in both of the approaches to analysis we adopted for the CPRD data. The use of a time-varying covariate analysis incorporates changes in exposure status throughout the follow-up period and thus is sensitive to picking up changes in risk of outcome which occur shortly after a change in exposure status [40]. This approach is supported by the recent EPIPHANY study findings which reported fatality percentages following a pulmonary embolism of 14% at 30 days and 27% at 90 days follow-up in 1033 cancer patients [41]. Therefore, the Landmark approach excludes a relatively high percentage of all VTE-related deaths and is more appropriate for assessing mortality longer term in patients who survive the initial event. The analysis also has some more favourable statistical properties as the alternative approach used (landmark analysis) does not include VTE events which occur after 6 months in addition to the exclusion of the 6-months following cancer diagnosis from the follow-up time However, this approach does have limitations especially when using routine healthcare data, as in the case of mortality as an outcome, acute medical events are more likely to get diagnosed in the intensive period of medical consultation which is known to take place in the weeks prior to death. In this particular context, however, the key advantage of the landmark approach is that it allows us to interpret how a VTE event occurring relatively soon after diagnosis (when the risk of VTE is highest) influences mortality longer term for which the clinical implications may be more apparent.

Finally we were unable to clearly establish whether factors such as cancer stage and underlying health status may have influenced the extent to which a VTE is associated with the risk of mortality. Whilst HRs were larger for women with local disease at the time of diagnosis, given that the risk of mortality was considerably higher in women with metastatic disease (314 deaths in 1200 person-years of follow-up) than in women with local disease (807 deaths in 32,000 person-years of follow-up) this is likely to be due to the issue of scale dependence whereby there is the potential for VTE to have a greater impact on a measure of relative association (such as the hazard ratio) in subgroups where the underlying risk of an outcome event is lower [42].

### Clinical implications

There are several mechanisms via which a VTE may exert a detrimental impact on cancer survival. There is an immediate impact due to the known high short-term fatality resulting from a thrombotic event which among all patients is estimated to be around 1% following a DVT and over 20% following a pulmonary embolism [41, 43]. Pooled results from two studies from the US and UK which would capture this short-term effect through incorporating VTE as a time-varying covariate indicate a greater than 2-fold of risk of mortality following a VTE. Compliance with existing clinical guidelines on primary prevention of VTE in cancer patients which advise targeting of prophylaxis in selected patients undergoing cancer surgery along with some patients in the outpatient setting [44–46]. However, it should be noted that in the Khorana score women with breast cancer may not be recommended for primary prophylaxis as these tend to score poorly on cancer type, anaemia and thrombocytosis. We have previously shown with this cohort that VTE events in women with breast cancer are likely to occur either during or immediately following chemotherapy or in the first month following surgery [6].

Cancer patients are at increased risk of bleeding from anticoagulation, with an estimated 2-fold increased risk for major bleeding compared to non-cancer patients [47]. Unsurprisingly, major and minor bleeding increases the hazard of death by over two-fold [48]. In addition, cancer patients are at 2–3 fold increased risk of recurrent VTE [47, 49–51]. However, based on the data from the current study, in the case where a woman with breast cancer is fortunate enough to survive her initial thrombotic event, the influence on long term prognosis is more difficult to establish, with a suggestion from this current study that mortality is not raised at all once cancer stage and underlying health status are taken into account. Guidelines from the UK National Institute of Health and Care Excellence (NICE) along with equivalent guidelines from other countries advise that cancer patients who develop VTE should receive at least 6 months of anticoagulation and in some instances treatment should continue indefinitely [52]. It is plausible to suggest that if adherence to these guidelines is good, then this could at least in part explain the relatively promising prognosis for women with breast cancer who survive their VTE, with prophylactic anticoagulation successfully mitigating against recurrent VTE (a likely cause of mortality). However the current NICE guidelines were not as robust in the era covered by the CPRD data and studies included in our meta-analysis. A move from vitamin-K antagonists to low molecular weight heparins in recent years because of greater efficacy in preventing recurrent VTE may further negate the negative survival impact of recurrent VTE [53]. More contemporary data reporting rates of VTE recurrence in cancer patients from the last decade as well as those with specific types of cancer are needed.

A further explanation for the detrimental impact of VTE on cancer survival relates to complex mechanisms underlying the symbiotic relationship between coagulation and tumour factors. Coagulation parameters are understood to play an important role in tumour progression and metastases, with changes in the haemostatic system evident in cancer patients even in the absence of a VTE [3]. It is hypothesized that VTE, even at the subclinical level of biochemical hypercoagulability, may have a role in promoting cancer growth and metastases and be associated with a more aggressive tumour behavior [54]. This has led researchers over many decades to explore the antineoplastic effects of anticoagulants and whether they could improve cancer survival even in the absence of a VTE. Overviews of the most recent randomized trial data comprising cancer patients without indication for anticoagulation (usually cancer outpatients) found no evidence of both oral anticoagulation (warfarin) [25] and low molecular weight heparin [50] on mortality at 12 months. However, evidence from the LMWH review indicates that this intervention does have modest (16%) reduction in longer term mortality, in line with previous evidence that the beneficial effects of LMWH occur after 12 months and also in patients with less advanced disease [51]. It is possible that if barriers to adherence with long term LMWH use could be overcome then there is the potential for a greater reduction mortality risks in cancer patients both with and without a previous VTE [55]. The current consensus is that future research in this area should focus on patients with specific cancer types rather than heterogeneous groups of tumours [3, 51].

## Conclusion

We report evidence that short-term mortality is raised in women with breast cancer following a VTE. However, when women are fortunate enough to survive their initial VTE event, the influence on mortality is far less certain due to considerable variability in results between individual studies. Future observational research on this topic should explore this heterogeneity by discovering whether there are specific groups of women with breast cancer for whom a VTE may exert a particularly poor prognostic effect, and for whom treatment strategies could therefore be influenced. Only with this knowledge along with more relevant data specific to women with breast cancer can we fully start to understand the true extent that deaths in breast cancer patients can be prevented by primary prophylaxis in those most at risk and whether presence of a VTE could influence cancer treatment strategies in these patients.

## Additional files

Additional file 1: Medline and EMBASE search strategies. Search strategies employed for the systematic review presented in the second part of the Results section. (DOCX 13 kb)

Additional file 2: Results from the CPRD (time-varying covariate analysis) stratified by comorbidity score (Charlson). Additional results where key results from the paper, difference in mortality risk between women with and without a VTE, were stratified by underlying health status as defined by Charlson comorbidity score. Results are presented for the analysis where VTE was treated as a time-varying covariate. (DOCX 14 kb)

Additional file 3: Influence of covariates on risk of mortality. Table showing the association with mortality for each of the covariates adjusted for in the CPRD analysis (non-time varying effect of VTE), with all other terms including VTE adjusted for. (DOCX 14 kb)

Additional file 4: Characteristics of Included Studies. Detailed overview of all studies included in the systematic review. (DOCX 23 kb)

## Abbreviations

CI: Confidence interval
CPRD: Clinical practice research datalink
DVT: Deep vein thrombosis
HES: Hospital episodes statistics
HR: Hazard ratio
ICD-10: 10th revision of the international statistical classification of diseases and related health problems
IQR: Interquartile range
NCIN: National cancer intelligence network
nTVA: non-time-varying analysis
ONS: Office of National Statistics
PE: Pulmonary embolism
RCT: Randomised-controlled trials
TVC: time-varying covariate analysis
VTE: Venous thromboembolism
