# Supplementary material for: Venous thromboembolism and mortality in breast cancer: cohort study with systematic review and meta-analysis
Source: BMC Cancer. 2017 Nov 10;17:747. doi: 10.1186/s12885-017-3719-1 (PMC5681811; doi:10.1186/s12885-017-3719-1)
Supplement: Supplementary file 1 — Medline and EMBASE search strategies. Search strategies employed for the systematic review presented in the second part of the Results section. (DOCX 13 kb) [file 12885_2017_3719_MOESM1_ESM.docx]

Appendix 1

MEDLINE search strategy

1. Epidemiological studies/

2. exp cohort studies/

3. (cohort adj (study or studies)).tw.

4. cohort analy$.tw.

5. (follow up adj (study or studies)).tw.

6. (observational adj (study or studies)).tw.

7. longitudinal.tw.

8. retrospective.tw.

9. prospective.tw.

10. incidence/

11. Risk/

12. 1 or 2 or 3 or 4 or 5 or 6 or 7 or 8 or 9 or 10 or 11

13. mortality.tw.

14. prognosis.tw.

15. death$.tw.

16. survival.tw.

17. mortality/

18. survival analysis/

19. prognosis/

20. death/

21. survival/

22. 13 or 14 or 15 or 16 or 17 or 18 or 19 or 20 or 21

23. exp venous thrombosis/

24. exp venous thromboembolism/

25. exp thrombosis/

26. exp pulmonary embolism/

27. dvt$.tw.

28. (deep adj8 (vein$ or ven$) adj8 (thromb$ or embol$)).tw.

29. 23 or 24 or 25 or 26 or 27 or 28

30. carcinoma/

31. malig$.tw.

32. neoplasm$.tw.

33. oncol$.tw.

34. tumo?r$.tw.

35. neoplasms/

36. exp breast neoplasm$/

37. cancer.ti.

38. 30 or 31 or 32 or 33 or 34 or 35 or 36 or 37

39. 12 and 22 and 29 and 38

1. limit 39 to (english language and humans)

EMBASE search strategy

1. epidemiology/

2. exp COHORT ANALYSIS/

3. (cohort adj (study or studies)).tw.

4. cohort analy$.tw.

5. (follow up adj (study or studies)).tw.

6. (observational adj (study or studies)).tw.

7. longitudinal.tw.

8. retrospective.tw.

9. prospective.tw.

10. incidence/

11. 1 or 2 or 3 or 4 or 5 or 6 or 7 or 8 or 9 or 10

12. mortality.tw.

13. prognosis.tw.

14. death$.tw.

15. survival.tw.

16. mortality/

17. survival/ or disease specific survival/ or life expectancy/ or long term survival/ or overall survival/ or survival rate/ or survival time/

18. standardized mortality ratio/

19. prognosis/

20. death/

21. 12 or 13 or 14 or 15 or 16 or 17 or 18 or 19 or 20

22. exp VEIN THROMBOSIS/

23. exp THROMBOSIS/

24. exp LUNG EMBOLISM/

25. exp VEIN EMBOLISM/

26. exp VENOUS THROMBOEMBOLISM/

27. exp DEEP VEIN THROMBOSIS/

28. dvt$.tw.

29. (deep adj8 (vein$ or ven$) adj8 (thromb$ or embol$)).tw.

30. 22 or 23 or 24 or 25 or 26 or 27 or 28 or 29

31. carcinoma/

32. malig$.tw.

33. neoplasm$.tw.

34. oncol$.tw.

35. tumo?r$.tw.

36. neoplasms/

37. exp BREAST TUMOR/

38. cancer.ti.

39. 31 or 32 or 33 or 34 or 35 or 36 or 37 or 38

40. 11 and 21 and 30 and 39

41. limit 40 to (human and english language)
