# Supplementary material for: Venous thromboembolism and mortality in breast cancer: cohort study with systematic review and meta-analysis
Source: BMC Cancer. 2017 Nov 10;17:747. doi: 10.1186/s12885-017-3719-1 (PMC5681811; doi:10.1186/s12885-017-3719-1)
Supplement: Supplementary file 2 — Results from the CPRD (time-varying covariate analysis) stratified by comorbidity score (Charlson). Additional results where key results from the paper, difference in mortality risk between women with and without a VTE, were stratified by underlying health status as defined by Charlson comorbidity score. Results are presented for the analysis where VTE was treated as a time-varying covariate. (DOCX 14 kb) [file 12885_2017_3719_MOESM2_ESM.docx]

Table S1: Results from the CPRD (time-varying covariate analysis) stratified by comorbidity score (Charlson)

|  | **Time-Varying (follow-up from cancer diagnosis)** | | | | | | | | | | | | |
| --- | --- | --- | --- | --- | --- | --- | --- | --- | --- | --- | --- | --- | --- |
|  | | |  |  | **Unadjusted** | | | **Adjusted for age** | | | **Multivariate Model*** | | |
|  |  |  | **No. of patients** | **No. Died** | **HR** | **95% CI** | | **HR** | **95% CI** | | **HR** | **95% CI** | |
| **All patients** | | **No VTE** | 12591 | 3504 | 1 |  |  | 1 |  |  | 1 |  |  |
|  |  | **VTE** | 611 | 298 | 2.97 | 2.62 | 3.36 | 2.58 | 2.27 | 2.92 | 2.42 | 2.13 | 2.75 |
| **Charlson score 0** | | **No VTE** | 6692 | 1795 | 1 |  |  | 1 |  |  | 1 |  |  |
|  |  | **VTE** | 295 | 129 | 3.20 | 2.67 | 3.82 | 2.70 | 2.26 | 3.23 | 2.33 | 1.94 | 2.80 |
| **Charlson score 1-3** | | **No VTE** | 5567 | 1579 | 1 |  |  | 1 |  |  | 1 |  |  |
|  |  | **VTE** | 293 | 124 | 2.84 | 2.36 | 3.41 | 2.55 | 2.12 | 3.06 | 2.59 | 2.15 | 3.12 |
| **Charlson score ≥4** | | **No VTE** | 332 | 163 | 1 |  |  | 1 |  |  | 1 |  |  |
|  |  | **VTE** | 23 | 12 | 1.64 | 0.91 | 2.95 | 1.75 | 0.97 | 3.17 | 3.24 | 1.67 | 6.30 |

*adjusted for age, stage at diagnosis, grade, tamoxifen therapy, smoking, body mass index, surgery and chemotherapy

In the time-varying analysis, no. died for the “No VTE” group represents the number of deaths in women who never developed VTE.
