# Supplementary material for: Venous thromboembolism and mortality in breast cancer: cohort study with systematic review and meta-analysis
Source: BMC Cancer. 2017 Nov 10;17:747. doi: 10.1186/s12885-017-3719-1 (PMC5681811; doi:10.1186/s12885-017-3719-1)
Supplement: Supplementary file 3 — Influence of covariates on risk of mortality. Table showing the association with mortality for each of the covariates adjusted for in the CPRD analysis (non-time varying effect of VTE), with all other terms including VTE adjusted for. (DOCX 14 kb) [file 12885_2017_3719_MOESM3_ESM.docx]

Table S2: Influence of covariates on risk of mortality

|  |  | HR | 95% CI | |
| --- | --- | --- | --- | --- |
| Venous thromboembolism^a^ | No | 1 |  |  |
|  | Yes | 1.22 | 0.93 | 1.60 |
| Stage | Local disease | 1 |  |  |
|  | Regional disease | 1.94 | 1.75 | 2.14 |
|  | metastases | 5.00 | 4.27 | 5.86 |
|  | Unknown | 1.82 | 1.67 | 2.00 |
| Grade | Well differentiated | 1 |  |  |
|  | Moderately differentiated | 1.55 | 1.34 | 1.79 |
|  | Poorly differentiated | 2.29 | 1.97 | 2.65 |
|  | Unknown | 1.66 | 1.43 | 1.92 |
| Charlson score | 0 | 1 |  |  |
|  | 1 to 3 | 0.85 | 0.79 | 0.91 |
|  | ≥4 | 1.35 | 1.12 | 1.62 |
| Hormone therapy^b^ | No | 1 |  |  |
|  | Yes | 0.70 | 0.64 | 0.77 |
| Surgery | No | 1 |  |  |
|  | Yes | 0.52 | 0.48 | 0.56 |
| Chemotherapy | No | 1 |  |  |
|  | Yes | 2.13 | 1.95 | 2.32 |
| Smoking (current) | No | 1 |  |  |
|  | Yes | 1.98 | 1.75 | 2.24 |
| Body mass index | Underweight (<19 kg/m^2^) | 1.46 | 1.12 | 1.90 |
|  | Ideal (19-24.9 kg/m^2^) | 1 |  |  |
|  | Overweight (25-29.9 kg/m^2^) | 0.92 | 0.82 | 1.03 |
|  | Obese (30-34.9 kg/m^2^) | 1.23 | 1.08 | 1.42 |
|  | Morbidly obese (≥35 kg/m^2^) | 1.31 | 1.07 | 1.61 |
|  | Missing | 1.30 | 1.18 | 1.42 |
| Age | (per year) | 1.06 | 1.05 | 1.06 |

a In the 6 months following a cancer diagnosis
b Considered a surrogate for oestrogen receptor positive breast cancer

HR Hazard ratio
