# Supplementary material for: Venous thromboembolism and mortality in breast cancer: cohort study with systematic review and meta-analysis
Source: BMC Cancer. 2017 Nov 10;17:747. doi: 10.1186/s12885-017-3719-1 (PMC5681811; doi:10.1186/s12885-017-3719-1)
Supplement: Supplementary file 4 — Characteristics of Included Studies. Detailed overview of all studies included in the systematic review. (DOCX 23 kb) [file 12885_2017_3719_MOESM4_ESM.docx]

Table S3: Characteristics of Included Studies

| **First Author (Year)** | **Country** | **Age^a^** | **Cohort size (VTE/control)** | **Exposure** | **When VTE occurred** | **Follow-up time (median/ max)** | **Adjusted for** | **% deaths at 2 years^n^** | |
| --- | --- | --- | --- | --- | --- | --- | --- | --- | --- |
|  |  |  |  |  |  |  |  | **VTE** | **Control** |
| Gross (2007)[36] | USA | 75^b^ | 26563 (102/26461) | DVT & PE | VTE first^c^ | NA | Age, race, cancer characteristics and treatment, comorbidity, socioeconomic status | Not stated | Not stated |
| Chew (2007)[35] | USA | 62 | 108,255 (1299/106956)^d^ | DVT & PE^e^ | Cancer first^f^ | 24 months (max) | Age, race, number of comorbidities, tumour histology | 31 | 19 |
| Jones (2009)[37] | UK (Scotland) | NA | 39597 (92/39262)^g^ | DVT & PE | VTE first^h^ | 60 months (max) | Age, period of diagnosis, deprivation decile | Not stated | Not stated |
| Paneesha (2009)[38] | UK (England & Wales) | 68.1^i^ | 116 (74.3/43.5)^j^ | DVT | Cancer first^k^ | 21 months (median) | Nil | 38 | 13 |
| Kirwan (2011)[9] | UK (England) | 52.1^l^ | 134 (13/121) | DVT | Cancer first^m^ | 24 months (median) | Age, stage | 23 | 25 |
| Reboucas (unpublished 2015) | Brazil | NA | 450 (225/225) | DVT & PE | Cancer first | 15.4 months (median) | Disease status, performance status | Not stated | Not stated |
| CPRD (unpublished 2015) | UK | 63.1 | 13202 (654/12548) | DVT & PE | Cancer first | 5.5 years (median) | Age, stage, grade, comorbidity, ER status, smoking, BMI, surgery, chemotherapy | 17 | 10 |
| Cesarman-Maus (unpublished 2015) | Mexico | 51 | 374 (53/321) | DVT | Cancer first | 26.2 (median) | Nil | 50 | 18 |

DVT deep vein thrombosis; PE pulmonary embolism; NA not available; HR hazard ratio
a Mean or median as specified in paper
b Average of all cancers included, there was no breakdown of age for breast cancer patients in the study

c Gross et al. “We defined a VTE as presenting concomitantly with a cancer diagnosis if the VTE diagnosis was made between 6 months prior and 1 month after the initial cancer diagnosis.”

d Based on 1.2% cumulative VTE incidence over 2 years

e Study excluded upper extremity DVT

f Chew et al. “Cox proportional hazard models were used to analyze the effect of specified risk factors on the outcomes of VTE or death within 2 years of cancer diagnosis”

g VTE number only includes events in the 0-6 months preceding breast cancer diagnosis as this was deemed to be most likely related to cancer. Other figures of VTE were excluded. The control represents the total number of patients with no VTE in the 5 years preceding cancer diagnosis

h Jones et al. “Cancers were classified as occurring after a VTE if they represented a first diagnosis of malignancy (excluding nonmelanomatous skin cancers) in the 5 years after a VTE.”

i Weighted average for the median age for all patients with VTE (66) and without VTE (69). There is no separate breakdown for breast cancer patients

j The figures were not clear from the paper. The numbers were estimated based on breast cancer patients comprising 19.6% of VTE and non-VTE cases in cancer patients

k Paneesha et al. “The presence of known malignancy (defined by ongoing treatment for cancer or metastatic disease) and type of malignancy detected at the time of thrombosis or subsequently during follow-up was documented in the database.”

l Weighted average of patients with advanced breast cancer (55.8); early breast cancer (51.3); Neoadjuvant breast cancer (46.1)

m Kirwan et al. “Duplex ultrasound imaging (DUI) was performed 1 month following commencement of chemotherapy or if patients became symptomatic

n estimated from Kaplan-Meier plots (where available) and from the raw data for the CPRD study
